# Real-World Hospitalization Outcomes with On-Line Hemodiafiltration Versus High-Flux Hemodialysis: A Retrospective, International Cohort Study

**DOI:** 10.2215/CJN.0000000955

**Published:** 2025-12-23

**Authors:** Yan Zhang, Anke Winter, Linda H. Ficociello, Belén Alejos Ferrera, Paola Carioni, Christian Apel, Otto Arkossy, Michael Anger, Robert Kossmann, Len A. Usvyat, Stefano Stuard

**Affiliations:** 1 Renal Research Institute, New York, New York; 2 Fresenius Medical Care Deutschland GmbH, Bad Homburg, Germany; 3 Fresenius Medical Care, Global Medical Office, Palazzo Pignano, Italy; 4 Fresenius Medical Care Holdings Inc, Waltham, Massachusetts

**Keywords:** hemodialysis, hospitalization

## Abstract

**Key Points:**

- Compared with high-flux hemodialysis, postdilution high volume hemodiafiltration was associated with a lower number of hospital admissions.
- Compared with high-flux hemodialysis, postdilution high volume hemodiafiltration was associated with reduced days spent in the hospital.

**Background:**

Patients with ESKD undergoing hemodialysis experience high rates of hospitalizations and mortality, partly due to the incomplete removal of some toxic uremic molecules. To improve outcomes, multiple modalities of kidney replacement therapy have been developed, including high-flux hemodialysis and on-line hemodiafiltration (HDF). Notably, on-line high-volume HDF (HV-HDF) has demonstrated mortality benefits over high-flux hemodialysis in some randomized trials.

**Methods:**

This retrospective cohort study evaluated hospitalization outcomes among in-center dialysis patients treated with HV-HDF and high-flux hemodialysis at Fresenius Medical Care NephroCare centers across Europe, the Middle East, and Africa between January 2019 and December 2022. Data were extracted from the European Clinical Database. The primary outcome was all-cause hospitalization; secondary outcomes included cause-specific hospitalizations. Negative binomial regression was used to estimate incidence rate ratios (IRRs) for hospital outcomes, incorporating inverse probability of treatment weighting to adjust for baseline differences between treatment groups.

**Results:**

A total of 71,669 patients were included, with 45% receiving hemodialysis and 55% receiving HDF. During the follow-up period, patients in the HDF group underwent a total of 12,741,453 HDF treatments, with a mean convection volume of 25.8 L (84% with CV≥23L). Compared with hemodialysis, treatment with HDF was associated with a lower incidence of both hospital admissions (adjusted IRR, 0.80; 95% confidence interval, 0.79 to 0.82) and days spent in the hospital (adjusted IRR, 0.80; 95% confidence interval, 0.78 to 0.82). These reductions were consistent across subgroups analyzed and across most major causes of hospitalization, including cardiovascular disease, infections, and fluid-related complications.

**Conclusions:**

In this large, real-world cohort spanning multiple regions and dialysis centers, HV-HDF was associated with significantly lower rates of both hospital admissions and days spent in the hospital compared with treatment with high-flux hemodialysis. These findings suggest that HV-HDF may have the potential to reduce morbidity in patients with ESKD.

## Visual Abstract

**Figure:**
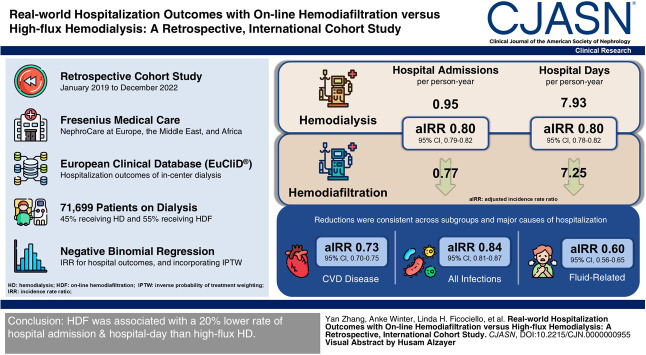

## Introduction

Patients on chronic hemodialysis bear a high burden of disease and are at a particularly high risk of mortality and hospitalization, with cardiovascular events and infection-related complications representing the greatest drivers for these events.^1–5^ Inpatient hospitalizations among patients with ESKD in the United States account for nearly half of total Medicare expenses in this patient population.^2,6,7^ Among other factors, the high burden of disease in these dialysis patients is attributed to incomplete removal of some toxic uremic molecules of medium and high-molecular mass.^8,9^ Multiple modalities of KRT have been developed to address these challenges. High-flux hemodialysis primarily uses diffusion to remove small uremic toxins from the blood and offers improved clearance of solutes in the middle mass range compared with low-flux hemodialysis.^8^ On-line hemodiafiltration (HDF) combines diffusion with convection to achieve superior clearance of middle and larger uremic toxins, including those involved in cardiovascular disease (CVD), chronic inflammation, and secondary immunodeficiency, while also enhancing effective removal of small toxins.^8–10^ On-line postdilution high-volume HDF (HV-HDF) is an intensive application of HDF, defined by a higher convection volume (CV) that aims to improve outcomes.^9,11^

Evidence surrounding the clinical benefit of HDF is evolving, and HDF has been linked to decreased cardiovascular damage, improved intradialytic hemodynamic stability, reduced inflammation, improved biocompatibility, and health-related quality of life.^9,12,13^ Several controlled trials and retrospective analyses comparing all-cause mortality and hospitalization outcomes have found conflicting results, with some studies demonstrating improved outcomes with HV-HDF.^11,14–16^ Most recently, the CONVective dialysis versus hemodialysis IN reducing mortality and Cardiovascular Events (CONVINCE) trial compared outcomes in 1360 ESKD patients and found that HV-HDF reduced all-cause mortality by 23% over a median follow-up of 30 months.^11^ Although these results are compelling, questions remain with respect to the generalizability of these outcomes to a broader patient population and their translatability to clinical practice, wherein convective volumes can vary significantly.^12,17,18^

To better understand the effects of HV-HDF in the context of a broader patient population and clinical outcomes, we conducted a retrospective cohort study to compare HV-HDF with high-flux hemodialysis on hospitalization burden in a large, unselected real-world hemodialysis population as well as across key demographic and clinical subgroups. Multiple hospitalization outcomes were accessed, including admissions, days spent in hospital, and cause-specific hospitalization—–end points that previous studies were generally underpowered to evaluate.

## Methods

### Study Design and Population

We conducted a retrospective cohort-study based on in-center dialysis patients treated in 659 Fresenius Medical Care Europe, Middle East, and Africa (EMEA) NephroCare centers from January 1, 2019, to December 31, 2022. All patients’ data were extracted from the European Clinical Database (EuCliD), a standardized electronic medical record system used in Fresenius Medical Care NephroCare clinics outside the United States.^19,20^ Information on race and ethnicity were extracted for countries where this information was collected. Adult patients (older than 18 years) were eligible for study inclusion if they granted permission for use of their pseudoanonymized data for secondary data analysis and had reported their initiation date of kidney replacement treatment. Patients were excluded if (*1*) they were from countries that did not systematically report coronavirus disease 2019 (COVID-19) cases during pandemic years (including the United Kingdom and Ireland) or (*2*) they were censored before their 91st day on hemodialysis.

This study was performed with adherence to the Declaration of Helsinki. The Ethics Committee of the Landesärztekammer Hessen (Medical Association of Hesse) in Frankfurt, Germany, reviewed and approved this study. All patients provided written informed consent for the secondary use of their data for scientific research purposes.

### Exposure Measures

Dialysis modality data were retrieved from each treatment in the EuCliD database. Only dialysis records of “on-line HDF” and “hemodialysis double needle” were counted as HDF and hemodialysis treatment, respectively. HDF and hemodialysis therapy were provided either by vascular access with two needles (graft or fistula) or by double lumen catheter. These modalities accounted for 99.1% of all treatments delivered across participating clinics. More than 98% of the sessions for hemodialysis treatment were delivered with high-flux dialyzer. All patients received treatments using ultrapure dialysate, and for those undergoing HV-HDF, the substitution fluid was sterile according to ISO 23500-5 2019 standards. Dialysis modality was defined based on the predominant type received during follow-up; patients were categorized as receiving HDF or hemodialysis if 75% or more of their dialysis sessions were conducted using HDF or hemodialysis, respectively.

COVID-19 infection status was determined by either a documented positive PCR severe acute respiratory syndrome coronavirus 2 test or International Classification of Diseases, Tenth Revision (ICD-10) codes suggestive of COVID-19 documented in the morbidity or mortality report (*i.e*., ICD-10 codes U07.1 and U07.2).

### Outcome Assessment

For each patient, the first treatment date during the study period was defined as the index date. All patients were followed up from the index date until death, kidney transplantation, modality change to peritoneal dialysis or home hemodialysis, spontaneous recovery, loss to follow-up (including dialysis center change out of NephroCare centers), or end of study period (December 31, 2022). Given the clinical instability commonly observed during the initial 90 days on dialysis, follow-up for patients who initiated dialysis < 90 days before study entry began on their 91st day. Information for all hospital admissions, including date of hospital admission, date of discharge, and cause of hospitalization, were extracted from the EuCliD database. The duration of each hospital stay was calculated as the time interval between hospital admission and discharge date, including both hospital admission and discharge dates. To ensure data quality and minimize the influence of outliers, the maximum duration of each individual hospital stay was capped at 30 days if the documentation in the database exceeded 30 days. The cumulative hospital stay across all hospital admissions per person during follow-up was defined as the “hospital days” outcome in our analysis. Causes of hospitalization were identified using ICD-10 codes, available in Supplemental Table 1, with attention given to the following causes: volume overload (including heart failure, fluid overload, pulmonary edema, and respiratory failure),^21^ CVD, infections including COVID-19 (hereafter referred to as “all infection”), and infections excluding COVID-19. Hospitalization from any cause was designated as the primary outcome, while cause-specific hospitalizations were designated as the secondary outcomes. The major cause of hospitalization was available in EuCliD for 90.3% of hospital records analyzed.

### Statistical Analysis

Summary statistics were calculated for patients who received HDF or hemodialysis based on demographic characteristics, kidney disease etiology, and comorbidities as of the index date. Dialysis vintage was calculated as the time from initiation of KRT until the index date. Vascular access was defined by the type of access used in > 75% of sessions over the past 6 months before the index date, if not available at the index date. Predialysis BP, blood flow rate, effective treatment time, body composition monitoring values, and laboratory measures were calculated as average values over the 6 months before the index date, if not available at the index date.

Inverse probability of treatment weighting (IPTW) was applied to balance patient baseline characteristics between the HDF and hemodialysis groups. First, the propensity scores—defined as the probability of being treated with HDF versus HD—were calculated using a logistic regression model. Covariates included demographic characteristics, kidney failure etiology, comorbidities, dialysis vintage, vascular access type, systolic BP, blood flow rate, and treatment time. Second, IPTW weights were calculated as the inverse of the propensity score for patients in the HDF group and the inverse of one minus the propensity score for those in the hemodialysis group. Covariate balance between the HDF and hemodialysis groups was assessed by the standardized differences before and after weighting. The average treatment effect of HDF relative to hemodialysis on the number of hospital admissions and total days hospitalized were estimated using IPTW-weighted negative binomial regression models, which accounted for overdispersion and multiple counts of the hospital outcomes. To reduce the effect of extreme weights on the variance of the effect estimate, stabilized IPTW was applied in the regression models by incorporating the marginal probability of treatment in the numerator of the weight calculation (*e.g*. proportion of HDF×IPTW). Robustness of the estimated associations was evaluated through multiple sensitivity analyses: (*1*) additional adjustment for strong confounders including age and vascular access type; (*2*) exclusion of patients from countries (Sweden, Czech Republic, Serbia, and Kyrgyzstan) where covariate balance remained suboptimal after weighting; and (*3*) by multivariable regression models adjusting for all covariates included in the propensity score calculation.

Subgroup analyses were performed for the primary outcome through stratifying patients by age (18–50 years, 50–65 years, and older than 65 years), sex, diabetes at index date, CVD history, dialysis vintage (≤1 years, >1–5 years, and >5 years), vascular access type (fistula, catheter), and COVID-19 status. To explore hospitalization causes potentially mitigated by HDF, multivariable negative binomial regression analyses were conducted for the secondary outcome, including hospitalization due to fluid-related events, CVD, and infection (including or excluding COVID-19). All analyses were performed using SAS statistical software version 9.4 (SAS Institute, Cary, NC).

## Results

### Patients

Of the 91,796 patients in the initial EuCliD dataset, a total of 71,669 patients met the study criteria and were included in the analysis (Supplemental Figure 1). From January 2019 to December 2022, 32,192 (45%) of these patients predominantly received hemodialysis and 39,477 (55%) patients received HDF (Table 1). The HDF patients received a total of 12,741,453 HDF treatments during follow-up, with a mean CV of 25.8 L (median [interquartile range, 25.9 [24.0–28.0]). At baseline, patients receiving HDF had a lower median age than those receiving hemodialysis (64.0 versus 67.0 years), a higher proportion of men (61% versus 58%), and a longer mean dialysis vintage (4.0 versus 3.4 years). The HDF group had a higher proportion of patients with fistula access (70% versus 55%), and a lower proportion of patients with catheter access (27% versus 44%) at baseline, but comparable blood flow rates (340.2 versus 333.3 ml/min). When examining comorbidities, the mean Charlson Comorbidity Index was similar across the two groups, while the HDF group had a higher percentage of patients with preexisting CVD (78% versus 74%) and a lower percentage of patients with preexisting diabetes (31% versus 36%). Other baseline characteristics were similar among the hemodialysis and HDF groups, including median effective treatment time and median blood flow rate. The standardized mean differences plot showed that all baseline characteristics were successfully balanced between HDF and hemodialysis groups after applying IPTW (Supplemental Figure 2). The median duration of follow-up was 22.6 (interquartile range, 9.3–43.5) months.

**Table 1:** Patient characteristics at baseline (*N*=71,669)

Baseline characteristics	HD (*N*=32,192)	HDF (*N*=39,477)
Age, yr, median (IQR)	67.0 (56.0–75.0)	64.0 (52.0–73.0)
Sex—Male, *n* (%)	18,737 (58)	24,199 (61)
**Ethnicity, *n* (%)**		
Caucasian	18,739 (58)	22,537 (57)
Other	538 (2)	3979 (10)
Unknown	12,915 (40)	12,961 (33)
**Smoking status, *n* (%)**		
Nonsmoker	14,477 (45)	19,193 (49)
Current/past smoker	7410 (23)	8475 (21)
Unknown	10,305 (32)	11,809 (30)
**Kidney failure etiology, *n* (%)**		
Diabetes mellitus	5241 (16)	5066 (13)
Hypertension	3687 (12)	4244 (11)
GN	3291 (10)	6839 (17)
Other causes	4899 (15)	7803 (20)
Unknown	15,074 (47)	15,525 (39)
Vintage in yr (mean±SD)	3.4±5.0	4.0±5.1
**Vascular access, *n* (%)**		
Fistula	17,656 (55)	27,524 (70)
Graft	452 (1)	1199 (3)
Catheter	14,057 (44)	10,688 (27)
Other	27 (0.1)	66 (0.2)
CCI, mean±SD	3.8±1.8	3.9±1.9
Preexisting diabetes, *n* (%)	11,707 (36)	12,182 (31)
Preexisting CVD, *n* (%)	23,936 (74)	30,887 (78)
BMI (kg/m^2^), mean±SD	26.7±6.1	27.1±6.0
Systolic BP (mm Hg), mean±SD	139.4±19.3	144.3±19.5
Diastolic BP (mm Hg), mean±SD	73.3±11.2	73.1±13.2
Dialysis frequency (day/wk), mean±SD	2.9±0.3	3.0±0.2
Effective treatment time (min), median (IQR)	241.1 (237.8–244.0)	241.7 (239.0–245.2)
Blood flow rate (ml/min), median (IQR)	333.3 (297.8–356.7)	340.2 (299.6–377.5)
Ultrafiltration volume (L), median (IQR)	2.1 (1.5–2.7)	2.2 (1.6–2.8)
**IDWG (Kg)**		
Missing (%)	3353 (10)	5408 (14)
Median (IQR)	1.8 (1.2–2.3)	1.9 (1.3–2.4)
**OCM Kt/V**		
Missing, n (%)	1773 (5)	1082 (3)
Mean±SD	1.5±0.3	1.6±0.4
**CV (L)**		
Median (IQR)	NA.	25.0 (22.5–27.1)

BMI, body mass index CCI, Charlson comorbidity index; CV, Convection volume; CVD, cardiovascular disease; HD, hemodialysis; HDF, hemodiafiltration; IDWG, interdialytic weight gain; IQR, interquartile range; NA, not applicable; OCM, online clearance monitoring.

### Rates of Hospital Admission and Hospital Days

**Figure 1:**
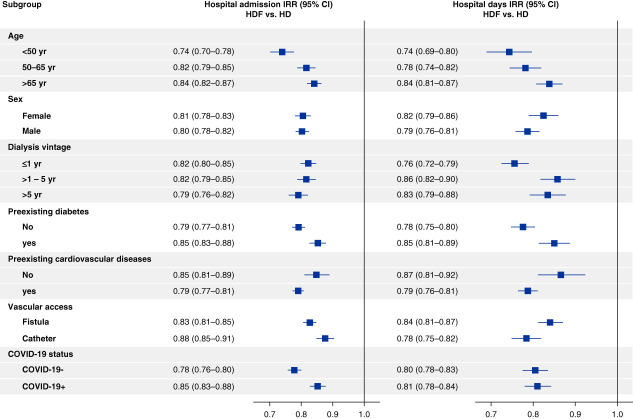
**IRR of hospital admission and hospital days among subgroups.** IRRs were estimated by negative binomial regression after applying IPTW; covariates included in IPTW are country, age, sex, ethnicity, tobacco use, kidney disease etiology, comorbidities (including diabetes, CVD, infectious disease, respiratory disease, digestive disease, genitourinary disease, and malignant disease), CCI, dialysis vintage, vascular access, systolic BP, blood flow rate, and effective treatment time at baseline. AVF, arteriovenous fistula; CCI, Charlson comorbidity index; CI, confidence interval; COVID-19, coronavirus disease 2019; HD, hemodialysis; HDF, hemodiafiltration; IPTW, inverse probability of treatment weighting; IRR, incidence rate ratio.

In the full cohort, the rates of hospital admissions and hospital days were numerically lower in the HDF group compared with the hemodialysis group (0.77 versus 0.95 admissions per person-year and 7.25 versus 7.93 days per person-year, respectively; Table 2). After controlling for confounding factors by IPTW, patients in the HDF group showed a 20% lower risk for both hospital admissions (adjusted incidence rate ratio [IRR], 0.80; 95% confidence interval [CI], 0.79 to 0.82) and hospital days (adjusted IRR, 0.80; 95% CI, 0.78 to 0.82). All sensitivity analyses, including additional adjustment for confounders in IPTW-weighted model and analysis conducted by multivariable regression, showed a similar lower risk for hospital admission, with IRRs ranging from 0.81 to 0.85 (Supplemental Table 2). A consistent pattern was also observed for hospital days, although the effect size was smaller in multivariable regression analyses. CV-stratified analyses showed that HDF patients with mean CV ≥25.8 L had further lower risk for both hospital admission (IRR, 0.70; 95% CI, 0.68 to 0.72) and hospital days (IRR, 0.74; 95% CI, 0.71 to 0.78) in IPTW-weighted model (Supplemental Table 3). A lower incidence of both hospital admissions and hospital days with HDF was consistently observed across various subgroups (Figure 1). The benefit of HDF was more pronounced for both rates of hospital admissions and hospital days among younger patients (younger than 50 years), patients free from diabetes at baseline, and patients with CVD.

**Table 2:** Association of dialysis modality with hospital admission and hospital days

Hospitalization Outcomes	HD	HDF
No. patients	32,192	39,477
No. hospital admission	58,777	67,888
Person-yr	61,688	87,869
Hospital admission rate (/person-yr)	0.95	0.77
Hospital days rate (/person-yr)	7.93	7.25
**Risk of hospital admission (IRR [95% CI])**		
Crude model	Reference	0.78 (0.77 to 0.80)
Stabilized IPTW^a^	Reference	0.80 (0.79 to 0.82)
**Risk of hospital days (IRR [95% CI**])		
Crude model	Reference	0.82 (0.80 to 0.85)
Stabilized IPTW^a^	Reference	0.80 (0.78 to 0.82)

CI, confidence interval; HD, hemodialysis; HDF, hemodiafiltration; IPTW, inverse probability of treatment weighting; IRR, incidence rate ratio.

a Incidence rate ratios were estimated by negative binomial regression after applying inverse probability of treatment weighting; covariates included in inverse probability of treatment weighting are country, age, gender, ethnicity, tobacco use, kidney disease etiology, comorbidities (including diabetes, cardiovascular disease, infectious disease, respiratory disease, digestive disease, genitourinary disease, and malignant disease), Charlson Comorbidity Index, dialysis vintage, vascular access, systolic BP, blood flow rate, and effective treatment time at baseline.

### Cause-Specific Hospital Admissions and Hospital Days

Of the investigated causes of interest, CVD was the most frequent cause of hospitalization overall (42.0% of all cases), followed by infections (35.1% all infections; 27.8% infections excluding COVID-19; Supplemental Figure 3). Fluid-related events accounted for 12.9% of hospitalizations (heart failure, 9.3%; fluid overload+pulmonary edema+respiratory failure, 3.6%).

**Figure 2:**
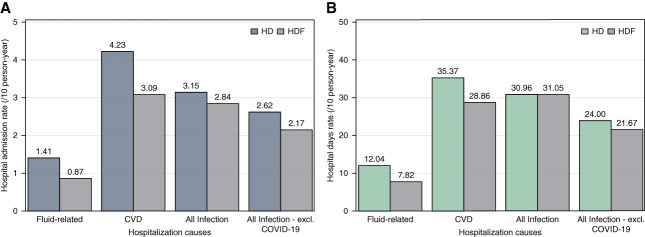
**Crude incidence rate of cause-specific hospital admission and hospital days.** (A) Incidence rate of cause-specific hospital admission by modality type (/10 person-year); (B) Incidence rate of cause-specific hospital days by modality type (days/10 person-year). CVD, cardiovascular disease.

**Figure 3:**
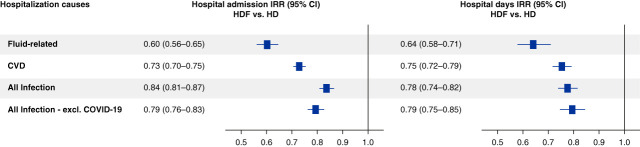
**IRR of cause-specific hospital admission and hospital days.** IRRs were estimated by negative binomial regression after applying IPTW; covariates included in IPTW are country, age, sex, ethnicity, tobacco use, kidney disease etiology, comorbidities (including diabetes, CVD, infectious disease, respiratory disease, digestive disease, genitourinary disease, and malignant disease), COVID-19, CCI, BMI, dialysis vintage, vascular access, systolic BP, blood flow rate, and effective treatment time at baseline. BMI, body mass index; CCI, Charlson comorbidity index; CI, confidence interval; HD, hemodialysis; HDF, hemodiafiltration; IPTW, inverse probability of treatment weighting; IRR, incidence rate ratio.

A crude data analysis indicated that both rates of hospital admissions and hospital days were lower with HDF versus hemodialysis for all causes of hospitalization analyzed, with the exception of hospital days due to all infections (Figure 2). Treatment with HDF was associated with lower incidence rates of hospital admissions and hospital days caused by CVD, all infections, infections excluding COVID-19, and fluid-related events compared to treatment with hemodialysis (Figure 3). The greatest effect was seen with the latter cause: the incidence rate of fluid-related hospital admissions in the HDF group was 40% lower and hospital days were 36% lower in comparison with hemodialysis (IRR, 0.60 [95% CI, 0.56 to 0.65] and IRR, 0.64 [95% CI, 0.58 to 0.71], respectively). HDF also had a notable effect on CVD-related hospitalizations, with incidence rates that were 27% lower for hospital admission and 25% lower for hospital days (IRR, 0.73; 95% CI, 0.70 to 0.75, and 0.75; 95% CI, 0.72 to 0.79, respectively). In addition, HDF was also associated with 21% lower incidence rate of infection-related hospital admissions (excluding COVID-19) and hospital days.

## Discussion

This large, retrospective study of 71,669 patients found that treatment with HDF was associated with fewer hospital admissions and days spent in the hospital relative to treatment with hemodialysis. This observation was consistent across various subgroups, including patients with diabetes at baseline, patients with CVD at baseline, and patients affected by COVID-19. Together with our previous study based on this cohort, demonstrating a 22% lower all-cause mortality and a 31% lower the risk of cardiovascular mortality with HDF compared with hemodialysis,^22^ these findings provide additional support for the possibility of clinical benefits from HDF in the real-world setting.

Data from previous studies characterizing the effect of HV-HDF on hospitalization outcomes are conflicting and largely derived from smaller, heavily selected clinical trial populations. In the CONVINCE trial, which enrolled 1360 patients as candidates for high CV (mean 25.3 L over follow-up) and with a relatively healthier baseline profile (including <22% coronary artery disease and <47% any CVD), no difference in recurrent all-cause and infection-related hospitalizations were observed.^11^ Null findings were also reported in two earlier studies with fewer than 800 patients and lower delivered convection/substitution volume (CONVective TRAnsport STudy trial, mean CV 20.7 L; Turkish Online-HDF trial, mean substitution volume 17.2 L).^23,24^ By contrast, the FRENCH Convective versus Hemodialysis In the Elderly trial (*N*=381) achieved gradually increasing CV from mean of 19.3 L at baseline to 22.5 L on the 24 months, and observed an 11% lower rate of all-cause hospital admissions (rate ratio [RR], 0.89; 95% CI, 0.76 to 1.04) and a 47% lower rate of vascular access–related admissions (RR, 0.53; 95% CI, 0.35 to 0.81) with HDF versus hemodialysis. The Estudio de Supervivencia de Hemodiafiltración OnLine trial (*N*=906; achieved mean CV 24 L) reported a 22% reduction in overall hospitalization with HDF (RR, 0.78; 95% CI, 0.67 to 0.90).^14^ Previous studies might be underpowered to detected difference in hospitalizations, particularly for cause-specific hospitalizations. In addition, none of these studies have evaluated the effect of HDF on total days hospitalized. Our study therefore adds a comprehensive, real-world assessment of HV-HDF versus hemodialysis on hospitalization burden.

Examining cause-specific hospitalizations may provide further insight into the treatment benefits offered by HDF. Our data demonstrated that HV-HDF was associated with lower rates of hospitalizations and hospital days driven by CVD, suggesting that HV-HDF may specifically affect cardiovascular health. Similarly, prior meta-analyses and trials have demonstrated that HV-HDF is associated with a lower risk of cardiovascular mortality.^14–16^ These improvements in cardiovascular outcomes are consistent with evidence that HV-HDF improves endothelial function and arterial stiffness by reducing oxidative stress and improving cardiovascular stability.^8,9^ Relatedly, it has been observed that greater interdialytic fluid retention in patients on hemodialysis is associated with higher all-cause and cardiovascular mortality and is thought to be a potential driver of cardiovascular events.^25,26^ One prospective study observed that volume overload in patients with CKD is associated with an increased rate of first hospitalization for congestive heart failures.^27^ Our study found that HV-HDF was associated with a reduced rate of fluid-related hospitalizations relative to hemodialysis. Decreased rates of heart failure-related and other CVD-related hospitalization outcomes with HV-HDF in this study may indicate improved volume and hemodynamic management with this treatment modality. Further exploring the relationship between HDF, fluid retention, and heart failure outcomes may be of clinical interest. HV-HDF was also associated with lower rates of hospitalizations and days spent in hospital due to all infections and infections excluding COVID-19 in our study. The Estudio de Supervivencia de Hemodiafiltración OnLine and CONVINCE trials similarly demonstrated a reduced risk of infection-related mortality with HDF versus hemodialysis.^11,14^ These findings, taken together, may indicate an immunomodulatory effect of HDF.

Currently, HDF is an established treatment option in Europe and Asia; however, hemodialysis is still the most used form of KRT globally.^13,18^ As evidence supporting the clinical advantages of HDF has been generated largely outside the United States, validation of comparable benefits of HDF over hemodialysis in broader populations, particularly within the United States, is necessary before evaluating further implications such as cost-effectiveness. Given the high-cost of hospitalizations among patients with ESKD in the United States,^2,6,7^ reducing hospitalization rates could yield meaningful savings to the health care system. Preliminary modeling by our group, which extrapolated reductions in hospitalizations observed with HV-HDF in the EMEA region to the US context, suggests the potential for sizable cost savings.^28^ However, extrapolation across regions need to additionally factor in patients and treatment level differences, such as lower arteriovenous fistula use, shorter treatment session, higher ultrafiltration rates, higher blood flow rates in the US compared with the EMEA.^29–32^ All these factors affect delivered CV, the key determinant of HDF efficacy.^33^ US-based studies are therefore warranted to provide a clear picture from both health and economic perspectives.

This study has limitations. Notably, baseline characteristics differed between the treatment modality groups; patients receiving HDF were, on average, younger, had a longer mean dialysis vintage, and had higher rates of preexisting CVD. Our statistical analyses accounted for these discrepancies by balancing the distribution of confounding variables with a propensity score weighting approach. However, owing to the retrospective nature of the study, there remains a risk of residual confounding or potential data misclassifications. Residual kidney function (RKF) was not captured in the database. Given the established association of RKF with hospitalization and mortality in dialysis patients,^34^ unmeasured differences in RKF between groups may have influenced our findings. The generalizability of these findings to populations outside the EMEA region, particularly the United States, may be limited due to differences in health care systems, dialysis practices, and patient demographics. Future across diverse geographic and clinical settings are needed to validate these findings and further explore the mechanisms underlying the observed benefits of HDF.

In summary, our multicenter retrospective cohort demonstrates HV-HDF was associated with fewer hospital admission and hospital days, particularly for cardiovascular, infection, and fluid-related causes, compared with high-flux hemodialysis. These findings add to a growing body of evidence suggesting potential benefits of HV-HDF. However, future research focused on broader populations (such as patients in the United States), and additional outcomes (including patient-reported outcomes and cost-effectiveness), as well as mechanistic studies on biologic pathways underlying the potential benefits of HDF are warranted. Together with cost-effectiveness analyses, such studies will help clarify the role of HDF and guide its optimal application across specific patient populations.

## Supplementary Material

## Data Availability

Original data generated for the study will be made available upon reasonable request to the corresponding author. Data Type: Statistical Analysis Plan; Software Source Code; Research Protocols; Health Care Data. Reason for Restricted Access: The datasets analyzed in the current study are not publicly available due to confidential protected patient information. Further inquiries can be addressed with the corresponding author.

## Supplemental Material

Supplemental Table 1. ICD-10 codes for cause of hospitalization.

Supplemental Table 2. Sensitivity analyses of association of dialysis modality with hospital admission and hospital days.

Supplemental Table 3. CV-specific associations of HDF with hospital admission and hospital days.

Supplemental Figure 1. Study flow chart.

Supplemental Figure 2. Standardized mean differences of covariates before and after weighting.

Supplemental Figure 3. Overall causes of hospitalization.
