# Supplementary material for: Real-World Hospitalization Outcomes with On-Line Hemodiafiltration Versus High-Flux Hemodialysis: A Retrospective, International Cohort Study
Source: Clin J Am Soc Nephrol. 2025 Dec 23;21(5):852–9. doi: 10.2215/CJN.0000000955 (PMC13143451; doi:10.2215/CJN.0000000955)
Supplement: Supplementary file 1 [file cjasn-21-852-s001.pdf]

ASN American Society of Nephrology · JASN Journal of the American Society of Nephrology · KIDNEY360 Accessing Our World From Every Angle · CJASN Clinical Journal of the American Society of Nephrology

ASN Journal Disclosure Form

As per ASN journal policy, I have disclosed any financial relationships or commitments I have held in the past 36 months as included below. I have listed my Current Employer below to indicate there is a relationship requiring disclosure. If no relationship exists, my Current Employer is not listed.

B. Alejos reports the following:
Employer: Fresenius Medical Care

I understand that the information above will be published within the journal article, if accepted, and that failure to comply and/or to accurately and completely report the potential financial conflicts of interest could lead to the following: 1) Prior to publication, article rejection, or 2) Post-publication, sanctions ranging from, but not limited to, issuing a correction, reporting the inaccurate information to the authors' institution, banning authors from submitting work to ASN journals for varying lengths of time, and/or retraction of the published work.

Name: Belen Alejos
Manuscript ID: CJASN-2025-001232
Manuscript Title: Real-world Hospitalization Outcomes with On-line Hemodiafiltration versus High-flux Hemodialysis: A Retrospective, International Cohort Study
Date of Completion: October 16, 2025
Disclosure Updated Date: October 16, 2025

ASN American Society of Nephrology · JASN Journal of the American Society of Nephrology · KIDNEY360 Accessing Our World From Every Angle · CJASN Clinical Journal of the American Society of Nephrology

ASN Journal Disclosure Form

As per ASN journal policy, I have disclosed any financial relationships or commitments I have held in the past 36 months as included below. I have listed my Current Employer below to indicate there is a relationship requiring disclosure. If no relationship exists, my Current Employer is not listed.

M. Anger reports the following:
Employer: Fresenius Medical Care; Ownership Interest: Pfizer; Fresenius Medical Care; Research Funding: Fresenius Medical Care; Advisory or Leadership Role: Fresenius Medical Care; and Speakers Bureau: Fresenius Medical Care.

I understand that the information above will be published within the journal article, if accepted, and that failure to comply and/or to accurately and completely report the potential financial conflicts of interest could lead to the following: 1) Prior to publication, article rejection, or 2) Post-publication, sanctions ranging from, but not limited to, issuing a correction, reporting the inaccurate information to the authors' institution, banning authors from submitting work to ASN journals for varying lengths of time, and/or retraction of the published work.

Name: Michael S. Anger
Manuscript ID: CJASN-2025-001232
Manuscript Title: Real-world Hospitalization Outcomes with On-line Hemodiafiltration versus High-flux Hemodialysis: A Retrospective, International Cohort Study
Date of Completion: October 10, 2025
Disclosure Updated Date: October 10, 2025

ASN American Society of Nephrology | JASN Journal of the American Society of Nephrology | KIDNEY360 Accessing Our World From Every Angle | CJASN Clinical Journal of the American Society of Nephrology

ASN Journal Disclosure Form

As per ASN journal policy, I have disclosed any financial relationships or commitments I have held in the past 36 months as included below. I have listed my Current Employer below to indicate there is a relationship requiring disclosure. If no relationship exists, my Current Employer is not listed.

C. Apel reports the following:
Employer: Fresenius Medical Care; and Ownership Interest: Fresenius Medical Care.

I understand that the information above will be published within the journal article, if accepted, and that failure to comply and/or to accurately and completely report the potential financial conflicts of interest could lead to the following: 1) Prior to publication, article rejection, or 2) Post-publication, sanctions ranging from, but not limited to, issuing a correction, reporting the inaccurate information to the authors' institution, banning authors from submitting work to ASN journals for varying lengths of time, and/or retraction of the published work.

Name: Christian Apel
Manuscript ID: CJASN-2025-001232
Manuscript Title: Real-world Hospitalization Outcomes with On-line Hemodiafiltration versus High-flux Hemodialysis: A Retrospective, International Cohort Study.
Date of Completion: October 15, 2025
Disclosure Updated Date: October 15, 2025

ASN Journal Disclosure Form

As per ASN journal policy, I have disclosed any financial relationships or commitments I have held in the past 36 months as included below. I have listed my Current Employer below to indicate there is a relationship requiring disclosure. If no relationship exists, my Current Employer is not listed.

O. Arkossy reports the following:
Employer: FMC; and Patents or Royalties: Fresenius Medical Care (FMC).

I understand that the information above will be published within the journal article, if accepted, and that failure to comply and/or to accurately and completely report the potential financial conflicts of interest could lead to the following: 1) Prior to publication, article rejection, or 2) Post-publication, sanctions ranging from, but not limited to, issuing a correction, reporting the inaccurate information to the authors' institution, banning authors from submitting work to ASN journals for varying lengths of time, and/or retraction of the published work.

Name: Otto Arkossy
Manuscript ID: CJASN-2025-001232
Manuscript Title: Real-world Hospitalization Outcomes with On-line Hemodiafiltration versus High-flux Hemodialysis: A Retrospective, International Cohort Study
Date of Completion: October 13, 2025
Disclosure Updated Date: October 13, 2025

ASN American Society of Nephrology JASN Journal of the American Society of Nephrology KIDNEY360 Accessing Our World From Every Angle CJASN Clinical Journal of the American Society of Nephrology

ASN Journal Disclosure Form

As per ASN journal policy, I have disclosed any financial relationships or commitments I have held in the past 36 months as included below. I have listed my Current Employer below to indicate there is a relationship requiring disclosure. If no relationship exists, my Current Employer is not listed.

P. Carioni reports the following:
Employer: Fresenius Medical Care Italia SpA

I understand that the information above will be published within the journal article, if accepted, and that failure to comply and/or to accurately and completely report the potential financial conflicts of interest could lead to the following: 1) Prior to publication, article rejection, or 2) Post-publication, sanctions ranging from, but not limited to, issuing a correction, reporting the inaccurate information to the authors' institution, banning authors from submitting work to ASN journals for varying lengths of time, and/or retraction of the published work.

Name: Paola Carioni
Manuscript ID: CJASN-2025-001232
Manuscript Title: Real-world Hospitalization Outcomes with On-line Hemodiafiltration versus High-flux Hemodialysis: A Retrospective, International Cohort Study
Date of Completion: October 13, 2025
Disclosure Updated Date: October 13, 2025

ASN
American Society of Nephrology
JASN
Journal of the American Society of Nephrology
KIDNEY360
Accessing Our World From Every Angle
CJASN
Clinical Journal of the American Society of Nephrology

ASN Journal Disclosure Form

As per ASN journal policy, I have disclosed any financial relationships or commitments I have held in the past 36 months as included below. I have listed my Current Employer below to indicate there is a relationship requiring disclosure. If no relationship exists, my Current Employer is not listed.

L. Ficociello reports the following:
Employer: Renal Research Institute, which is a wholly owned subsidiary of Fresenius Medical Care; and
Ownership Interest: Fresenius Medical Care.

I understand that the information above will be published within the journal article, if accepted, and that failure to comply and/or to accurately and completely report the potential financial conflicts of interest could lead to the following: 1) Prior to publication, article rejection, or 2) Post-publication, sanctions ranging from, but not limited to, issuing a correction, reporting the inaccurate information to the authors' institution, banning authors from submitting work to ASN journals for varying lengths of time, and/or retraction of the published work.

Name: Linda Ficociello
Manuscript ID: CJASN-2025-001232
Manuscript Title: Real-world Hospitalization Outcomes with On-line Hemodiafiltration versus High-flux Hemodialysis: A Retrospective, International Cohort Study
Date of Completion: October 15, 2025
Disclosure Updated Date: October 15, 2025

ASN American Society of Nephrology JASN Journal of the American Society of Nephrology KIDNEY360 Accessing Our World From Every Angle CJASN Clinical Journal of the American Society of Nephrology

ASN Journal Disclosure Form

As per ASN journal policy, I have disclosed any financial relationships or commitments I have held in the past 36 months as included below. I have listed my Current Employer below to indicate there is a relationship requiring disclosure. If no relationship exists, my Current Employer is not listed.

R. Kossmann reports the following:
Employer: Fresenius Medical Care; Ownership Interest: Fresenius Medical Care; Advisory or Leadership Role: Yes. I am an Executive Vice President and the Global Head of Medical Affairs for Fresenius Medical Care.; and Other Interests or Relationships: Fresenius Medical Care - full time employment.; Renal Physicians Association - I am a past president.

I understand that the information above will be published within the journal article, if accepted, and that failure to comply and/or to accurately and completely report the potential financial conflicts of interest could lead to the following: 1) Prior to publication, article rejection, or 2) Post-publication, sanctions ranging from, but not limited to, issuing a correction, reporting the inaccurate information to the authors' institution, banning authors from submitting work to ASN journals for varying lengths of time, and/or retraction of the published work.

Name: Robert J. Kossmann
Manuscript ID: CJASN-2025-001232
Manuscript Title: Real-world Hospitalization Outcomes with On-line Hemodiafiltration versus High-flux Hemodialysis: A Retrospective, International Cohort Study
Date of Completion: October 10, 2025
Disclosure Updated Date: October 10, 2025

ASN American Society of Nephrology
JASN Journal of the American Society of Nephrology
KIDNEY360 Accessing Our World From Every Angle
CJASN Clinical Journal of the American Society of Nephrology

ASN Journal Disclosure Form

As per ASN journal policy, I have disclosed any financial relationships or commitments I have held in the past 36 months as included below. I have listed my Current Employer below to indicate there is a relationship requiring disclosure. If no relationship exists, my Current Employer is not listed.

S. Stuard reports the following:
Employer: Fresenius Medical Care; Ownership Interest: Fresenius Medical Care; and Patents or Royalties: Fresenius Medical Care.

I understand that the information above will be published within the journal article, if accepted, and that failure to comply and/or to accurately and completely report the potential financial conflicts of interest could lead to the following: 1) Prior to publication, article rejection, or 2) Post-publication, sanctions ranging from, but not limited to, issuing a correction, reporting the inaccurate information to the authors' institution, banning authors from submitting work to ASN journals for varying lengths of time, and/or retraction of the published work.

Name: Stefano Stuard
Manuscript ID: CJASN-2025-001232
Manuscript Title: Real-world Hospitalization Outcomes with On-line Hemodiafiltration versus High-flux Hemodialysis: A Retrospective, International Cohort Study
Date of Completion: October 11, 2025
Disclosure Updated Date: October 11, 2025

ASN American Society of Nephrology | JASN Journal of the American Society of Nephrology | KIDNEY360 Accessing Our World From Every Angle | CJASN Clinical Journal of the American Society of Nephrology

ASN Journal Disclosure Form

As per ASN journal policy, I have disclosed any financial relationships or commitments I have held in the past 36 months as included below. I have listed my Current Employer below to indicate there is a relationship requiring disclosure. If no relationship exists, my Current Employer is not listed.

L. Usvyat reports the following:
Employer: Renal Research Institute; Ownership Interest: Fresenius Medical Care; and Advisory or Leadership Role: Privacy Analytics board.

I understand that the information above will be published within the journal article, if accepted, and that failure to comply and/or to accurately and completely report the potential financial conflicts of interest could lead to the following: 1) Prior to publication, article rejection, or 2) Post-publication, sanctions ranging from, but not limited to, issuing a correction, reporting the inaccurate information to the authors' institution, banning authors from submitting work to ASN journals for varying lengths of time, and/or retraction of the published work.

Name: Len A. Usvyat
Manuscript ID: CJASN-2025-001232R1
Manuscript Title: Real-world Hospitalization Outcomes with On-line Hemodiafiltration versus High-flux Hemodialysis: A Retrospective, International Cohort Study
Date of Completion: November 13, 2025
Disclosure Updated Date: October 28, 2025

ASN American Society of Nephrology | JASN Journal of the American Society of Nephrology | KIDNEY360 Accessing Our World From Every Angle | CJASN Clinical Journal of the American Society of Nephrology

ASN Journal Disclosure Form

As per ASN journal policy, I have disclosed any financial relationships or commitments I have held in the past 36 months as included below. I have listed my Current Employer below to indicate there is a relationship requiring disclosure. If no relationship exists, my Current Employer is not listed.

A. Winter reports the following:
Employer: Fresenius Medical Care Deutschland GmbH

I understand that the information above will be published within the journal article, if accepted, and that failure to comply and/or to accurately and completely report the potential financial conflicts of interest could lead to the following: 1) Prior to publication, article rejection, or 2) Post-publication, sanctions ranging from, but not limited to, issuing a correction, reporting the inaccurate information to the authors' institution, banning authors from submitting work to ASN journals for varying lengths of time, and/or retraction of the published work.

Name: Anke Winter
Manuscript ID: CJASN-2025-001232
Manuscript Title: Real-world Hospitalization Outcomes with On-line Hemodiafiltration versus High-flux Hemodialysis: A Retrospective, International Cohort Study
Date of Completion: October 13, 2025
Disclosure Updated Date: October 13, 2025

ASN American Society of Nephrology | JASN Journal of the American Society of Nephrology | KIDNEY360 Accessing Our World From Every Angle | CJASN Clinical Journal of the American Society of Nephrology

ASN Journal Disclosure Form

As per ASN journal policy, I have disclosed any financial relationships or commitments I have held in the past 36 months as included below. I have listed my Current Employer below to indicate there is a relationship requiring disclosure. If no relationship exists, my Current Employer is not listed.

Y. Zhang reports the following:
Employer: Fresenius Medical Care

I understand that the information above will be published within the journal article, if accepted, and that failure to comply and/or to accurately and completely report the potential financial conflicts of interest could lead to the following: 1) Prior to publication, article rejection, or 2) Post-publication, sanctions ranging from, but not limited to, issuing a correction, reporting the inaccurate information to the authors' institution, banning authors from submitting work to ASN journals for varying lengths of time, and/or retraction of the published work.

Name: Yan Zhang
Manuscript ID: CJASN-2025-001232
Manuscript Title: Real-world Hospitalization Outcomes with On-line Hemodiafiltration versus High-flux Hemodialysis: A Retrospective, International Cohort Study
Date of Completion: October 9, 2025
Disclosure Updated Date: October 9, 2025
