# Supplementary material for: Real-World Hospitalization Outcomes with On-Line Hemodiafiltration Versus High-Flux Hemodialysis: A Retrospective, International Cohort Study
Source: Clin J Am Soc Nephrol. 2025 Dec 23;21(5):852–9. doi: 10.2215/CJN.0000000955 (PMC13143451; doi:10.2215/CJN.0000000955)
Supplement: Supplementary file 2 [file cjasn-21-852-s002.pdf]

SUPPLEMENTAL MATERIAL

Table of contents

Supplemental Table 1. International Classification of Diseases, 10th Revision codes for cause of hospitalization

Supplemental Table 2. Sensitivity analyses of association of dialysis modality with hospital admission and hospital days

Supplemental Table 3. Convection volume specific associations of HDF with hospital admission and hospital days

Supplemental Figure 1. Study flowchart

Supplemental Figure 2. Standardized mean differences of covariates before and after weighting

Supplemental Figure 3. Overall causes of hospitalization.

SUPPLEMENTAL MATERIAL

Supplemental Table 1. International Classification of Diseases, 10th Revision codes for cause of hospitalization

**Volume overload codes**
**Heart failure**<br>I11.0, I13.0, I13.1, I13.2, I27, I27.0, I27.1, I27.2, I27.8, I27.9, I42.0, I42.1, I42.2, I42.3, I42.4, I42.5, I42.6, I42.7, I42.8, I42.9, I43, I43.0, I43.0*, I43.1, I43.1*, I43.2, I43.2*, I43.8, I43.8*, I50, I50.0, I50.1, I50.2, I50.3, I50.4, I50.9, I51, I51.7, K76.1, P29.0<br>**Fluid overload**<br>E87.7, R06.0, R60, R60.0, R60.1, R60.9, T70.4<br>**Pulmonary edema**<br>E87.7, J81<br>**Respiratory failure**<br>J96, J96.0, J96.1, J96.2, J96.9
**Cardiovascular disease codes**
E10.5, E11.5, E12.5, E13.5, E14.5, G45, G45.0, G45.1, G45.2, G45.3, G45.4, G45.8, G45.9, G46, G46.0, G46.1, G46.2, G46.3, G46.4, G46.5, G46.6, G46.7, G46.8, H34, H34.0, H34.1, H34.2, H34.8, H34.9, I00, I01, I01.0, I01.1, I01.2, I01.8, I01.9, I02.0, I05, I05.0, I05.1, I05.2, I05.8, I05.9, I06, I06.0, I06.1, I06.2, I06.8, I06.9, I07, I07.0, I07.1, I07.2, I07.8, I07.9, I08, I08.0, I08.1, I08.2, I08.3, I08.8, I08.9, I09, I09.0, I09.1, I09.2, I09.8, I09.9, I10, I11, I11.0, I11.9, I12, I12.0, I12.9, I13, I13.0, I13.1, I13.2, I13.9, I15, I15.0, I15.1, I15.2, I15.8, I15.9, I20, I20.0, I20.1, I20.8, I20.9, I21, I21.0, I21.1, I21.2, I21.3, I21.4, I21.9, I22, I22.0, I22.1, I22.8, I22.9, I23, I23.0, I23.1, I23.2, I23.3, I23.4, I23.5, I23.6, I23.8, I24, I24.0, I24.1, I24.8, I24.9, I25, I25.0, I25.1, I25.2, I25.3, I25.4, I25.5, I25.6, I25.8, I25.9, I26, I26.0, I26.9, I27, I27.0, I27.1, I27.2, I27.8, I27.9, I28, I28.0, I28.1, I28.8, I28.9, I30, I30.0, I30.1, I30.8, I30.9, I31, I31.0, I31.1, I31.2, I31.3, I31.8, I31.9, I32, I32.0, I32.1, I32.8, I33, I33.0, I33.9, I34, I34.0, I34.1, I34.2, I34.8, I34.9, I35, I35.0, I35.1, I35.2, I35.8, I35.9, I36, I36.0, I36.1, I36.2, I36.8, I36.9, I37, I37.0, I37.1, I37.2, I37.8, I37.9, I38, I39, I39.0, I39.1, I39.2, I39.3, I39.4, I39.8, I40, I40.0, I40.1, I40.8, I40.9, I41, I41.0, I41.1, I41.2, I41.8, I42, I42.0, I42.1, I42.2, I42.3, I42.4, I42.5, I42.6, I42.7, I42.8, I42.9, I43, I43.0, I43.1, I43.2, I43.8, I44, I44.0, I44.1, I44.2, I44.3, I44.4, I44.5, I44.6, I44.7, I45, I45.0, I45.1, I45.2, I45.3, I45.4, I45.5, I45.6, I45.8, I45.9, I46, I46.0, I46.1, I46.9, I47, I47.0, I47.1, I47.2, I47.9, I48, I48.0, I48.1, I48.2, I48.3, I48.4, I48.9, I49, I49.0, I49.1, I49.2, I49.3, I49.4, I49.5, I49.8, I49.9, I50, I50.0, I50.1, I50.9, I51, I51.0, I51.1, I51.2, I51.3, I51.4, I51.5, I51.6, I51.7, I51.8, I51.9, I52, I52.0, I52.1, I52.8, I60, I60.0, I60.1, I60.2, I60.3, I60.4, I60.5, I60.6, I60.7, I60.8, I60.9, I61, I61.0, I61.1, I61.2, I61.3, I61.4, I61.5, I61.6, I61.8, I61.9, I62, I62.0, I62.1, I62.9, I63, I63.0, I63.1, I63.2, I63.3, I63.4, I63.5, I63.6, I63.8, I63.9, I64, I65, I65.0, I65.1, I65.2, I65.3, I65.8, I65.9, I66, I66.0, I66.1, I66.2, I66.3, I66.4, I66.8, I66.9, I67, I67.0, I67.1, I67.2, I67.3, I67.4, I67.5, I67.6, I67.7, I67.8, I67.9, I68, I68.0, I68.1, I68.2, I68.8, I69, I69.0, I69.1, I69.2, I69.3, I69.4, I69.8, I70, I70.0, I70.1, I70.2, I70.8, I70.9, I71, I71.0, I71.1, I71.2, I71.3, I71.4, I71.5, I71.6, I71.8, I71.9, I72, I72.0, I72.1, I72.2, I72.3, I72.4, I72.5, I72.6, I72.8, I72.9, I73, I73.0, I73.1, I73.8, I73.9, I74, I74.0, I74.1, I74.2, I74.3, I74.4, I74.5, I74.8, I74.9, I77, I77.0, I77.1, I77.2, I77.3, I77.4, I77.5, I77.6, I77.8, I77.9, I78, I78.0, I78.1, I78.8, I78.9, I79, I79.0, I79.1, I79.2, I79.8, I80, I80.0, I80.1, I80.2, I80.3, I80.8, I80.9, I81, I82, I82.0, I82.1, I82.2, I82.3, I82.8, I82.9, I83, I83.0, I83.1, I83.2, I83.9, I85, I85.0, I85.9, I86, I86.0, I86.1, I86.2, I86.3, I86.4, I86.8, I87, I87.0, I87.1, I87.2, I87.8, I87.9, I88, I88.0, I88.1, I88.8, I88.9, I89, I89.0, I89.1, I89.8, I89.9, I95, I95.0, I95.1, I95.2, I95.8, I95.9, I97, I97.0, I97.1, I97.2, I97.8, I97.9,

I98, I98.0, I98.1, I98.2, I98.3, I98.8, I99, K76.1, K76.2, M31.8, M31.9, O22.5, O22.8, O22.9, O87.3, O87.8, O87.9, O90.3, O99.4, P29.0, R00.0, R00.1, R00.2, T81.7
**COVID-19 codes**
U07.1, U07.2
**Infection codes**
A00, A00.0, A00.1, A00.9, A01, A01.0, A01.1, A01.2, A01.3, A01.4, A02, A02.0, A02.1, A02.2, A02.8, A02.9, A03, A03.0, A03.1, A03.2, A03.3, A03.8, A03.9, A04, A04.0, A04.1, A04.2, A04.3, A04.4, A04.5, A04.6, A04.7, A04.8, A04.9, A05, A05.0, A05.1, A05.2, A05.3, A05.4, A05.8, A05.9, A06, A06.0, A06.1, A06.2, A06.3, A06.4, A06.5, A06.5†, A06.6, A06.6†, A06.7, A06.8, A06.9, A07, A07.0, A07.1, A07.2, A07.3, A07.8, A07.9, A08, A08.0, A08.1, A08.2, A08.3, A08.4, A08.5, A09, A09.0, A09.9, A15, A15.0, A15.1, A15.2, A15.3, A15.4, A15.5, A15.6, A15.7, A15.8, A15.9, A16, A16.0, A16.1, A16.2, A16.3, A16.4, A16.5, A16.7, A16.8, A16.9, A17, A17.0, A17.0†, A17.1, A17.1†, A17.8, A17.8†, A17.9, A17.9†, A18, A18.0, A18.0†, A18.1, A18.2, A18.3, A18.4, A18.5, A18.6, A18.7, A18.7†, A18.8, A19, A19.0, A19.1, A19.2, A19.8, A19.9, A20, A20.0, A20.1, A20.2, A20.3, A20.7, A20.8, A20.9, A21, A21.0, A21.1, A21.2, A21.3, A21.7, A21.8, A21.9, A22, A22.0, A22.1, A22.2, A22.7, A22.8, A22.9, A23, A23.0, A23.1, A23.2, A23.3, A23.8, A23.9, A24, A24.0, A24.1, A24.2, A24.3, A24.4, A25, A25.0, A25.1, A25.9, A26, A26.0, A26.7, A26.8, A26.9, A27, A27.0, A27.8, A27.9, A28, A28.0, A28.1, A28.2, A28.8, A28.9, A30, A30.0, A30.1, A30.2, A30.3, A30.4, A30.5, A30.8, A30.9, A31, A31.0, A31.1, A31.8, A31.9, A32, A32.0, A32.1, A32.1†, A32.7, A32.8, A32.9, A33, A34, A35, A36, A36.0, A36.1, A36.2, A36.3, A36.8, A36.9, A37, A37.0, A37.1, A37.8, A37.9, A38, A39, A39.0, A39.1, A39.2, A39.3, A39.4, A39.5, A39.8, A39.9, A40, A40.0, A40.1, A40.2, A40.3, A40.8, A40.9, A41, A41.0, A41.1, A41.2, A41.3, A41.4, A41.5, A41.8, A41.9, A42, A42.0, A42.1, A42.2, A42.7, A42.8, A42.9, A43, A43.0, A43.1, A43.8, A43.9, A44, A44.0, A44.1, A44.8, A44.9, A46, A48, A48.0, A48.1, A48.2, A48.3, A48.4, A48.8, A49, A49.0, A49.1, A49.2, A49.3, A49.8, A49.9, A50, A50.0, A50.1, A50.2, A50.3, A50.4, A50.5, A50.6, A50.7, A50.9, A51, A51.0, A51.1, A51.2, A51.3, A51.4, A51.5, A51.9, A52, A52.0, A52.0†, A52.1, A52.2, A52.3, A52.7, A52.8, A52.9, A53, A53.0, A53.9, A54, A54.0, A54.1, A54.2, A54.3, A54.4, A54.4†, A54.5, A54.6, A54.8, A54.9, A55, A56, A56.0, A56.1, A56.2, A56.3, A56.4, A56.8, A57, A58, A59, A59.0, A59.8, A59.9, A6.0†, A6.1†, A6.5†, A60, A60.0, A60.1, A60.9, A63, A63.0, A63.8, A64, A65, A66, A66.0, A66.1, A66.2, A66.3, A66.4, A66.5, A66.6, A66.7, A66.8, A66.9, A67, A67.0, A67.1, A67.2, A67.3, A67.9, A68, A68.0, A68.1, A68.9, A69, A69.0, A69.1, A69.2, A69.8, A69.9, A70, A71, A71.0, A71.1, A71.9, A74, A74.0, A74.0†, A74.8, A74.9, A75, A75.0, A75.1, A75.2, A75.3, A75.9, A77, A77.0, A77.1, A77.2, A77.3, A77.8, A77.9, A78, A79, A79.0, A79.1, A79.8, A79.9, A80, A80.0, A80.1, A80.2, A80.3, A80.4, A80.9, A81, A81.0, A81.1, A81.2, A81.8, A81.9, A82, A82.0, A82.1, A82.9, A83, A83.0, A83.1, A83.2, A83.3, A83.4, A83.5, A83.6, A83.8, A83.9, A84, A84.0, A84.1, A84.8, A84.9, A85, A85.0, A85.0†, A85.1, A85.1†, A85.2, A85.8, A86, A87, A87.0, A87.0†, A87.1, A87.1†, A87.2, A87.8, A87.9, A88, A88.0, A88.1, A88.8, A89, A92, A92.0, A92.1, A92.2, A92.3, A92.4, A92.5, A92.8, A92.9, A93, A93.0, A93.1, A93.2, A93.8, A94, A95, A95.0, A95.1, A95.9, A96, A96.0, A96.1, A96.2, A96.8, A96.9, A97, A97.0, A97.1, A97.2, A97.9, A98, A98.0, A98.1, A98.2, A98.3, A98.4, A98.5, A98.8, A99, B00, B00.0, B00.1, B00.2, B00.3, B00.3†, B00.4, B00.4†, B00.5, B00.7, B00.8, B00.9, B01, B01.0, B01.0†, B01.1, B01.1†, B01.2, B01.2†, B01.8, B01.9, B02, B02.0, B02.0†, B02.1, B02.1†, B02.2, B02.2†, B02.3, B02.7, B02.8, B02.9, B03, B04, B05, B05.0, B05.0†, B05.1, B05.1†, B05.2, B05.2†, B05.3, B05.3†, B05.4, B05.8, B05.9, B06, B06.0, B06.0†, B06.8, B06.9, B07, B08, B08.0, B08.1, B08.2, B08.3, B08.4, B08.5, B08.8, B09, B15, B15.0, B15.9, B16, B16.0, B16.1, B16.2, B16.9, B17, B17.0, B17.1, B17.2, B17.8, B17.9, B18, B18.0, B18.1, B18.2, B18.8, B18.9, B19, B19.0, B19.9, B20, B20.0, B20.1, B20.2, B20.3, B20.4, B20.5, B20.6, B20.7,

B20.8, B20.9, B21, B21.0, B21.1, B21.2, B21.3, B21.7, B21.8, B21.9, B22, B22.0, B22.1, B22.2, B22.7, B23, B23.0, B23.1, B23.2, B23.8, B24, B25, B25.0, B25.0†, B25.1, B25.1†, B25.2, B25.2†, B25.8, B25.9, B26, B26.0, B26.0†, B26.1, B26.1†, B26.2, B26.2†, B26.3, B26.3†, B26.8, B26.9, B27, B27.0, B27.1, B27.8, B27.9, B30, B30.0, B30.0†, B30.1, B30.1†, B30.2, B30.3, B30.3†, B30.8, B30.8†, B30.9, B33, B33.0, B33.1, B33.2, B33.3, B33.4, B33.4†, B33.8, B34, B34.0, B34.1, B34.2, B34.3, B34.4, B34.8, B34.9, B35, B35.0, B35.1, B35.2, B35.3, B35.4, B35.5, B35.6, B35.8, B35.9, B36, B36.0, B36.1, B36.2, B36.3, B36.8, B36.9, B37, B37.0, B37.1, B37.2, B37.3, B37.3†, B37.4, B37.5, B37.5†, B37.6, B37.6†, B37.7, B37.8, B37.9, B38, B38.0, B38.1, B38.2, B38.3, B38.4, B38.4†, B38.7, B38.8, B38.9, B39, B39.0, B39.1, B39.2, B39.3, B39.4, B39.5, B39.9, B40, B40.0, B40.1, B40.2, B40.3, B40.7, B40.8, B40.9, B41, B41.0, B41.7, B41.8, B41.9, B42, B42.0, B42.0†, B42.1, B42.7, B42.8, B42.9, B43, B43.0, B43.1, B43.2, B43.8, B43.9, B44, B44.0, B44.1, B44.2, B44.7, B44.8, B44.9, B45, B45.0, B45.1, B45.2, B45.3, B45.7, B45.8, B45.9, B46, B46.0, B46.1, B46.2, B46.3, B46.4, B46.5, B46.8, B46.9, B47, B47.0, B47.1, B47.9, B48, B48.0, B48.1, B48.2, B48.3, B48.4, B48.5, B48.7, B48.8, B49, B50, B50.0, B50.8, B50.9, B51, B51.0, B51.8, B51.9, B52, B52.0, B52.8, B52.9, B53, B53.0, B53.1, B53.8, B54, B55, B55.0, B55.1, B55.2, B55.9, B56, B56.0, B56.1, B56.9, B57, B57.0, B57.0†, B57.1, B57.2, B57.3, B57.4, B57.5, B58, B58.0, B58.0†, B58.1, B58.1†, B58.2, B58.2†, B58.3, B58.3†, B58.8, B58.9, B60, B60.0, B60.1, B60.2, B60.8, B64, B65, B65.0, B65.1, B65.2, B65.3, B65.8, B65.9, B66, B66.0, B66.1, B66.2, B66.3, B66.4, B66.5, B66.8, B66.9, B67, B67.0, B67.1, B67.2, B67.3, B67.4, B67.5, B67.6, B67.7, B67.8, B67.9, B68, B68.0, B68.1, B68.9, B69, B69.0, B69.1, B69.8, B69.9, B70, B70.0, B70.1, B71, B71.0, B71.1, B71.8, B71.9, B72, B73, B74, B74.0, B74.1, B74.2, B74.3, B74.4, B74.8, B74.9, B75, B76, B76.0, B76.1, B76.8, B76.9, B77, B77.0, B77.8, B77.9, B78, B78.0, B78.1, B78.7, B78.9, B79, B80, B81, B81.0, B81.1, B81.2, B81.3, B81.4, B81.8, B82, B82.0, B82.9, B83, B83.0, B83.1, B83.2, B83.3, B83.4, B83.8, B83.9, B85, B85.0, B85.1, B85.2, B85.3, B85.4, B86, B87, B87.0, B87.1, B87.2, B87.3, B87.4, B87.8, B87.9, B88, B88.0, B88.1, B88.2, B88.3, B88.8, B88.9, B89, B90, B90.0, B90.1, B90.2, B90.8, B90.9, B91, B92, B94, B94.0, B94.1, B94.2, B94.8, B94.9, B95, B95.0, B95.1, B95.2, B95.3, B95.4, B95.5, B95.6, B95.7, B95.8, B95.9, B96, B96.0, B96.1, B96.2, B96.3, B96.4, B96.5, B96.6, B96.7, B96.8, B97, B97.0, B97.1, B97.2, B97.3, B97.4, B97.5, B97.6, B97.7, B97.8, B98, B98.0, B98.1, B99, C02.0, D86, D86.0, D86.1, D86.2, D86.3, D86.8, D86.9, E32.1, F02.8, F02.8*, G00, G00.0, G00.1, G00.2, G00.3, G00.8, G00.9, G01, G02, G02.0, G02.0*, G02.1, G02.1*, G02.8, G02.8*, G03, G03.0, G03.1, G03.8, G03.9, G04, G04.0, G04.2, G04.8, G04.9, G05, G05.0, G05.0*, G05.1, G05.1*, G05.2, G05.2*, G05.8, G05.8*, G06, G06.0, G06.1, G06.2, G07, G08, G09, G14, G37.4, G92, H00, H00.0, H00.1, H01.0, H02.3, H10, H10.0, H10.1, H10.2, H10.3, H10.4, H10.5, H10.8, H10.9, H32.0, H32.0*, H60.0, H60.1, H60.2, H60.3, H66, H66.0, H66.1, H66.2, H66.3, H66.4, H67.0, H67.0*, H67.1, H67.1*, H67.8, H67.8*, H70.0, H70.1, H70.2, H75.0, H75.0*, H83.0, I00, I01, I01.0, I01.1, I01.2, I01.8, I01.9, I02, I02.0, I02.9, I05, I06, I06.0, I06.1, I06.2, I06.8, I09.0, I09.1, I09.2, I09.8, I09.9, I30.1, I32, I32.0, I32.0*, I32.1, I32.1*, I32.8, I32.8*, I33.0, I40.0, I41, J00, J01, J01.0, J01.1, J01.2, J01.3, J01.4, J01.8, J01.9, J02, J02.0, J02.8, J02.9, J03, J03.0, J03.8, J03.9, J04, J04.0, J04.1, J04.2, J05, J05.0, J05.1, J06, J06.0, J06.8, J06.9, J09, J10, J10.0, J10.1, J10.8, J11, J11.0, J11.1, J11.8, J12, J12.0, J12.1, J12.2, J12.3, J12.8, J12.9, J13, J14, J15, J15.0, J15.1, J15.2, J15.3, J15.4, J15.5, J15.6, J15.7, J15.8, J15.9, J16, J16.0, J16.8, J17, J17.0, J17.0*, J17.1, J17.1*, J17.2, J17.2*, J17.3, J17.3*, J17.8, J17.8*, J18, J18.0, J18.1, J18.2, J18.8, J18.9, J20, J20.0, J20.1, J20.2, J20.3, J20.4, J20.5, J20.6, J20.7, J20.8, J20.9, J21, J21.0, J21.1, J21.8, J21.9, J22, J31, J31.0, J31.1, J31.2, J32, J32.0, J32.1, J32.2, J32.3, J32.4, J32.8, J34.0, J35, J35.0, J36, J37, J37.0, J37.1, J39.0, J39.1, J40, J41, J41.0, J41.1, J41.8, J42, J85, J85.0, J85.1, J85.2, J85.3, J86, J86.0, J86.9, K04.6, K04.7, K11.3, K12.2, K35.2, K35.3, K35.8, K36, K37, K50.0, K50.1, K50.8, K50.9, K51.0, K51.2, K51.3, K51.4, K51.8, K51.9, K57.0, K57.1, K57.2, K57.3, K57.4, K57.5, K57.8, K57.9, K61, K61.0, K61.1, K61.2, K61.3, K61.4, K63.0, K65, K65.0, K65.8, K67, K67.0, K67.0*, K67.1, K67.1*, K67.2, K67.2*, K67.3, K67.3*, K67.8, K67.8*, K75.0, K75.1, K81.0, K81.1, K81.9, L00, L01,

L02, L02.0, L02.1, L02.2, L02.3, L02.4, L02.8, L02.9, L03, L03.0, L03.1, L03.2, L03.3, L03.8, L03.9, L04, L04.0, L04.1, L04.2, L04.3, L04.8, L04.9, L05, L05.0, L05.9, L08, L08.0, L08.1, L08.8, L08.9, L44.4, L70.2, L88, L92.8, L94.6, L98.0, L98.3, M00, M00.0, M00.1, M00.2, M00.8, M00.9, M01, M01.0, M01.1, M01.2, M01.3, M01.4, M01.5, M01.6, M01.8, M02.1, M02.3, M02.6, M35.2, M46.2, M46.3, M46.6, M60.0, M71.0, M86.0, M86.1, M86.2, M86.3, M86.4, M86.5, M86.6, M86.8, M86.9, M90.0, M90.1, M90.2, M90.3, N10, N11.0, N11.1, N12, N13.6, N15.1, N30, N30.0, N34.0, N34.1, N34.2, N34.3, N35.1, N37, N41.0, N41.1, N41.2, N41.3, N45, N45.0, N45.9, N48.1, N49.0, N49.1, N49.2, N49.8, N61, N70, N70.0, N71.0, N71.1, N72, N73.0, N73.1, N73.2, N73.3, N73.4, N73.5, N74, N75.1, N76.0, N76.1, N76.2, N76.3, N76.4, N77.1, N77.1*, N98.0, O03.0, O03.5, O04.5, O05.0, O05.5, O06.0, O06.5, O07.0, O07.5, O08.0, O23, O23.0, O23.1, O23.2, O23.3, O23.4, O23.5, O23.9, O41.1, O75.3, O86, O86.0, O86.1, O86.2, O86.3, O86.8, O91, O91.0, O91.1, O98, O98.0, O98.1, O98.2, O98.3, O98.4, O98.5, O98.6, O98.7, O98.8, O98.9, P23, P23.0, P23.1, P23.2, P23.3, P23.4, P23.5, P23.6, P23.8, P23.9, P35, P35.0, P35.1, P35.2, P35.3, P35.4, P35.8, P35.9, P36, P36.0, P36.1, P36.2, P36.3, P36.4, P36.5, P36.8, P36.9, P37, P37.0, P37.1, P37.2, P37.3, P37.4, P37.5, P37.8, P37.9, P38, P39, P39.0, P39.1, P39.2, P39.3, P39.4, P39.8, P39.9, P58.2, R75, T79.3, T80.2, T81.4, T82.6, T82.7, T83.5, T83.6, T84.5, T84.6, T84.7, T85.7, T87.4, T88.0, U07.1, U07.2, U60.1, U60.2, U60.3, U60.9, U61.1, U61.2, U61.3, U61.9, Z11.4, Z20.2, Z20.5, Z20.6, Z21, Z24.6, Z71.7, Z83.0

Supplemental Table 2. Sensitivity analyses of association of dialysis modality with hospital admission and hospital days

Regression model	IRR (95% CI)	
	Hospital admission	Hospital days
Stabilized IPTW* + age + vascular access	0.85 (0.84 – 0.87)	0.81 (0.78 – 0.82)
Stabilized IPTW* (in selected countries**)	0.81 (0.79 – 0.82)	0.80 (0.78 – 0.82)
IPTW*	0.82 (0.80 – 0.83)	0.81 (0.79 – 0.83)
Multivariable regression – Model 1†	0.85 (0.83 – 0.87)	0.92 (0.89 – 0.95)
Multivariable regression – Model 2‡	0.83 (0.81 – 0.85)	0.89 (0.86 – 0.93)

*Covariates included in IPTW are country, age, gender, ethnicity, tobacco use, renal etiology, comorbidities (including diabetes, cardiovascular disease, infectious disease, respiratory disease, digestive disease, genitourinary disease, malignant disease), Charlson Comorbidity Index, dialysis vintage, vascular access, systolic blood pressure, blood flow rate, effective treatment time at baseline.

**Excluding patients from Sweden, Czech Republic, Serbia, and Kyrgyzstan showing not well balance after inverse probability weighting.

†Model 1. Adjusted for age, gender, ethnicity, tobacco use, renal etiology, comorbidities (including diabetes, cardiovascular disease, infectious disease, respiratory disease, digestive disease, genitourinary disease, malignant disease), Charlson Comorbidity Index, dialysis vintage, vascular access, systolic blood pressure, blood flow rate, and effective treatment time at baseline.

‡Model 2. Similar to Model 1 and additionally adjusted for parameters calculated as the average values over the past 6 months prior to baseline (if not available at baseline), including IDWG, treatment frequency, OCM Kt/V, overhydration, albumin, sodium, calcium, iPTH, hemoglobin, platelets, leukocytes.

IDWG, interdialytic weight gain; iPTH, serum intact parathyroid hormone; IPTW, Inverse probability of treatment weighting; IRR, incidence rate ratio, OCM, online clearance monitoring.

Supplemental Table 3. Convection volume specific associations of HDF with hospital admission and hospital days

	HDF (mean CV < 25.8 L) versus HD	HDF (mean CV ≥ 25.8 L) versus HD
Risk of hospital admission (IRR [95% CI])		
Stabilized IPTW*	0.92 (0.90–0.94)	0.70 (0.68–0.72)
Stabilized IPTW* + age + vascular access	0.94 (0.92–0.96)	0.75 (0.73–0.77)
Risk of hospital days (IRR [95% CI])		
Stabilized IPTW*	0.89 (0.86–0.92)	0.74 (0.71–0.76)
Stabilized IPTW* + age + vascular access	0.90 (0.87–0.93)	0.76 (0.73–0.78)

*IRRs were estimated by negative binomial regression after applying IPTW; covariates included in IPTW are country, age, gender, ethnicity, tobacco use, renal etiology, comorbidities (including diabetes, cardiovascular disease, infectious disease, respiratory disease, digestive disease, genitourinary disease, malignant disease), Charlson Comorbidity Index, dialysis vintage, vascular access, systolic blood pressure, blood flow rate, and effective treatment time at baseline.

CI, confidence interval; CV, convection volume, HD, hemodialysis; HDF, hemodiafiltration; IPTW, inverse probability of treatment weighting; IRR, incidence rate ratio.

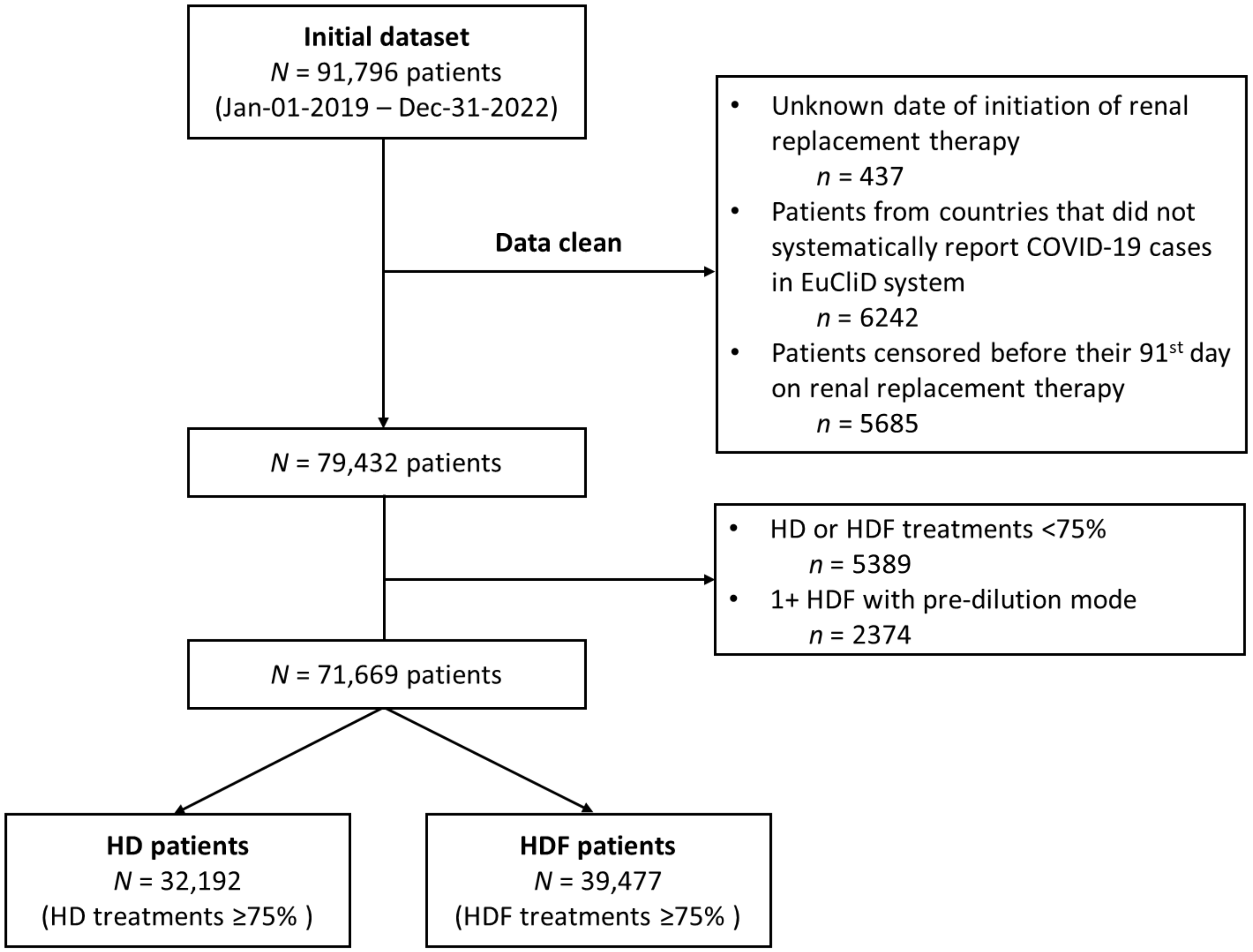

Supplemental Figure 1. Study flowchart.
COVID-19, coronavirus disease 2019; EuCliD, European Clinical Database; HD, hemodialysis; HDF, hemodiafiltration.

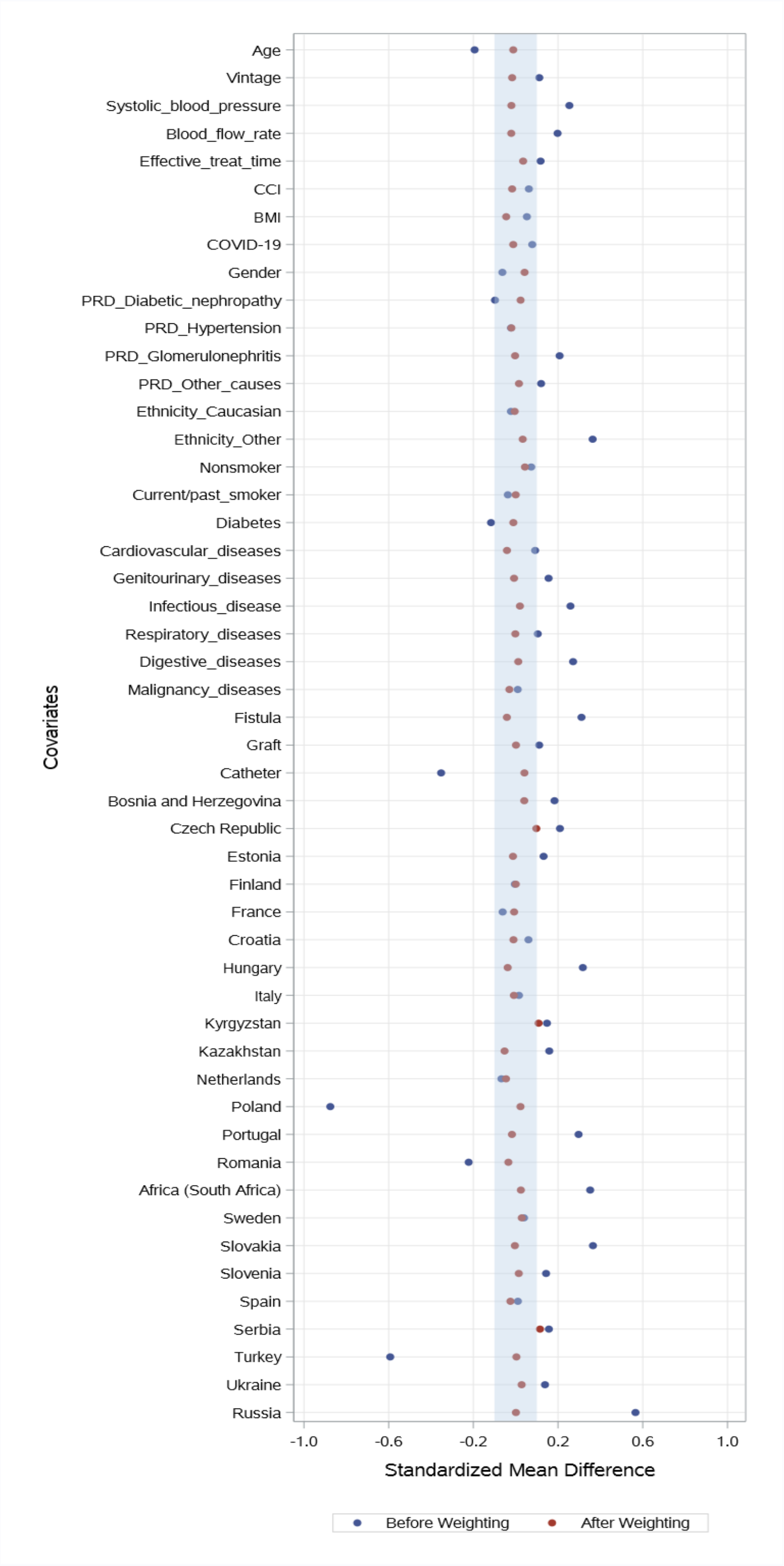

Supplemental Figure 2. Standardized mean differences of covariates before and after weighting. CCI, Charlson Comorbidity Index; COVID-19, coronavirus disease 2019; PRD; Primary renal disease.

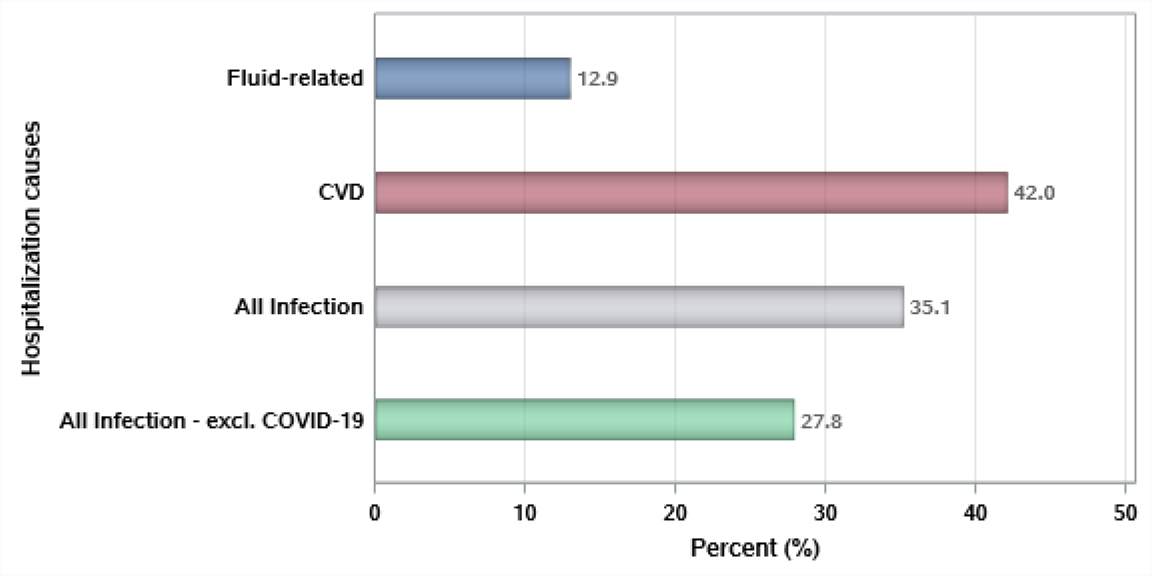

Supplemental Figure 3. Overall causes of hospitalization.
CVD, cardiovascular disease; COVID-19, coronavirus disease 2019.
